# Exploring the Dynamic Relationship Between Migraine and Functional Neurological Disorders: A Narrative Review

**DOI:** 10.1007/s11916-026-01480-w

**Published:** 2026-04-13

**Authors:** Tiffany Eatz, Jason Margolesky, Alexandra Cocores

**Affiliations:** University of Miami Miller School of Medicine, Miami, Fl USA

**Keywords:** Headache, Migraine, Functional Neurologic Disorder, Functional Movement Disorder, Psychogenic non-epileptic seizures, Comorbidity

## Abstract

**Purpose of Review:**

Headache and functional neurologic disorders (FND) are the most diagnosed neurologic disorders, yet their relationship has not been comprehensively detailed.

**Recent Findings:**

Understandings of migraine and FND have each progressed tremendously. Headache may be the most common neurologic comorbidity in FND. Migraine may be identified as a predisposing factor and predate FND up to ten years. Schemas have been proposed to explain potential relationship and shared pathophysiologic mechanisms; the disorders share multiple comorbidities. Headache may contribute to disability in FND. Treatment of migraine may improve functional symptoms; both entities respond to multidisciplinary approaches.

**Summary:**

Migraine and FND commonly coexist, contributing to overall disability and decreased quality of life (QOL). Pathophysiologic interactions may impact management and outcomes. Headache history should be obtained in FND patients; headache when present should be treated appropriately. Future research should evaluate FND in cohorts of headache patients and if headache treatment could improve symptoms and QOL for specific FND phenotypes.

## Introduction

Headache disorders and functional neurologic disorders (FND) are the two most encountered diagnoses in new patient referrals to neurology outpatient clinics (headache 19% and FND 16%) [1]. These distinct entities each may carry considerable economic burden and healthcare resource utilization [2–4]. High proportions of patients with either of these disorders report significant stigma, which can negatively affect quality of life and outcomes [5, 6].

In this narrative review, we synthesize the current literature pertaining to the epidemiology, pathophysiology, and clinical manifestations of headache and FND. A recent systemic review and meta-analysis evaluated pain in FND, but did not include studies which described headache disorders [7]. In this narrative review, we focus on what is known regarding the clinical features and diagnosis of headache disorders among patients with specific FND, summarize current understanding of management, and highlight knowledge gaps.

### Epidemiology and Burden

#### Migraine and Headache Disorders

Approximately 2.81 billion individuals worldwide have migraine or tension-type headache, with females aged 15–49 years being most affected. Peak incidence of migraine occurs at ages 20–24 years in women (18.2/1000 person-years) and ages 15–19 years in men (6.2/1000 person-years). Cumulative incidence is 43% in women and 18% in men [8]. The one-year prevalence is 17.1% in women and 5.6% in men, peaking in midlife and lower in adolescence and older age [9].

Migraine is associated with substantial decrements in health-related quality of life [10] and can negatively affect many important aspects of life including interpersonal relationships and financial achievement [11]. Headache disorders are the third most common cause of years lived with a disability across all ages and sexes combined [12].

#### Functional Neurologic Disorders

After headache, functional symptoms are the second most common reason to see a neurologist [1]. Up to one-third of new neurology outpatient adults are thought to have symptoms that are not all or only somewhat ‘explained by organic disease,’ most of whom are eventually diagnosed with FND in isolation or in combination with another neurological disease [13].

Comprehensive epidemiological investigations are lacking, however estimated incidence is 4–12/100,000/year [14]. FND disproportionately affects women (3:1) although as age of onset increases, the proportion of men affected increases [15]. For PNES, the estimated incidence is 1.4–4.9/100,000/year and prevalence 2–33/100,000 [16]. A recent meta-analysis revealed that functional movement disorders (FMD) peak in midlife for all phenotypes and prevalence is 2–3 times higher in women than in men [15].

FND are associated with worse physical and mental health status and higher rates of disability [17]. There is a substantial direct and indirect economic cost of FND, including excess utilization of healthcare resources [18]. A recent economic analysis revealed costs of FND care surpass those of similarly complex neurologic conditions [19]. The experience of patients with FND reflects unmet needs below standards for long-term neurological conditions [20].

#### Historical Perspectives

**Fig. 1:**
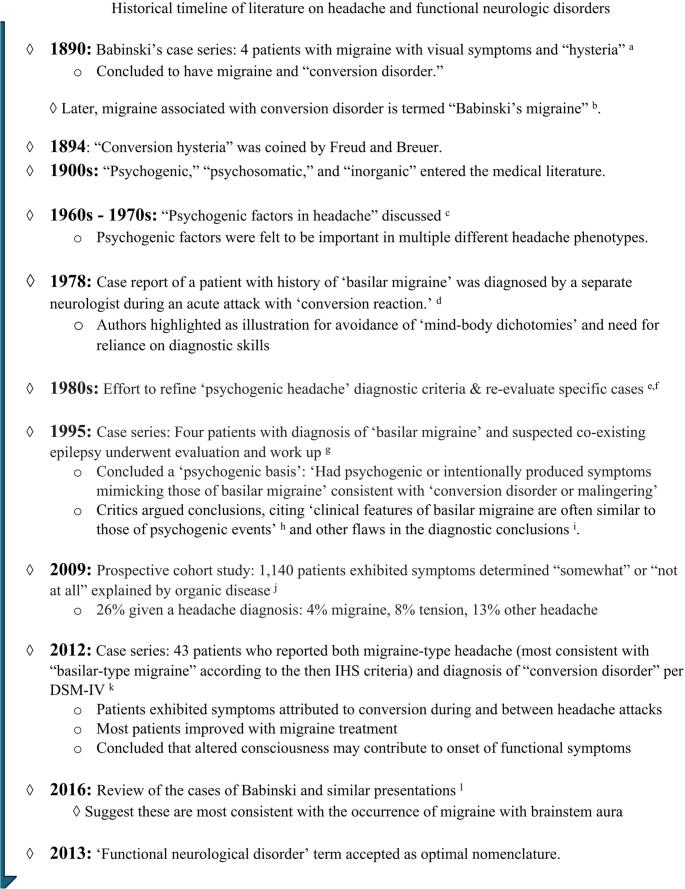
Historical timeline of literature on headache and functional neurologic disorders

Migraine headache disorder and FND are both highly stigmatized conditions. This phenomenon may be a result of the poor understanding of these disorders historically. Early literature on the co-occurrence and possible relationship between functional symptoms and headache symptoms is sparse, although what is available does highlight the significant advancements made over time in understanding these conditions (Fig. 1).

The prior historical idea of migraine driven by psychiatric alterations and neurotic traits is now fundamentally shifted by our knowledge that it is a disorder of the brain with neurovascular etiology. Similarly, the prior concepts to explain FND have been refined: initial concepts of FND date to ancient times, when symptoms were attributed to supernatural phenomenon. Eventually, the cognitive and neurocircuitry models were elucidated, with patients exhibiting cognitive biases toward bodily symptoms [31].

Both the nomenclature for and the clinical approach to these sets of disorders has evolved over time. The understanding of both headache disorders and FND have progressed tremendously. There is now more well-defined and evidence-based understanding of the pathophysiologic process for each of these disorders, and their diagnostic criteria has been clearly defined.

## Diagnosis

The diagnosis for both primary headache disorders and FND is made clinically (Table 1) through detailed medical history and neurologic examination.

**Table 1:** Diagnostic criteria for migraine headache disorder and functional neurologic disorder

**Migraine headache disorder** – ICHD-3 diagnostic criteria:
A. At least five attacks fulfilling criteria B-D
B. Headache lasting 4-72 hours (untreated or unsuccessfully treated)
C. Headache has at least two of the following:
a. Unilateral location
b. Pulsating quality
c. Moderate or severe pain intensity
d. Aggravation by or causing avoidance of routine physical activity
D. During headache at least one of the following:
a. Nausea and/or vomiting
b. Photophobia and phonophobia
E. Not better accounted by another ICHD-3 diagnosis.
**Functional Neurological Symptoms Disorder** – DSM-5 Diagnostic Criteria
A. The patient has ≥ 1 symptoms of altered voluntary motor or sensory function.
B. Clinical findings provide evidence of incompatibility between the symptom and recognized neurological or medical conditions.
C. The symptom or deficit is not better explained by another medical or mental disorder.
D. The symptom or deficit causes clinically significant distress or impairment in social, occupational, or other important areas of functioning or warrants medical evaluation.

The International Classification of Headache disorders (ICHD), now in its third version, provides diagnostic criteria for headache disorders [21]. Primary headache disorders are diagnosed clinically, without required radiologic or laboratory testing. If a patient’s history or examination indicated suspicion for a secondary headache disorder, testing may be indicated for diagnosis.

FND are diagnosed according to the American Psychiatric Association’s Diagnostic and Statistical Manual of Mental Disorders (DSM), now in its fifth edition [22]. The clinical diagnosis is founded on the diagnostic pillars of inconsistency of symptoms over time and during the exam as well as incongruence of the symptoms with understood neuroanatomy and neuro-pathophysiology. Patients may present with a myriad of neurologic symptoms or deficits that can morph over time. Challenging clinical scenarios may raise the need to explore differential diagnoses with host of conditions; however, FND are not a diagnosis of exclusion, and no specific testing may be required if positive symptoms are elicited on exam.

### Headache Attributed to Somatization Disorder

Diagnosis The ICHD-3 defines ‘headache attributed to somatization disorder’ as a secondary headache disorder attributed to psychiatric disorder [21]. Diagnostic criteria are based on the DSM-IV diagnosis, which is not included in the latest version. The diagnosis requires at least four pain symptoms and multiple non-pain symptoms (at least two gastrointestinal, one sexual, and one neurologic). The headache must have evidence of causation demonstrated by its time course in parallel with the other functional symptoms.

### Pathophysiology

#### Migraine Headache Disorder

Migraine is a sensory processing disorder with a genetic basis [23], the manifestations of which may be influenced by environmental factors, comorbid medical conditions, and other individual risk factors [24]. Migraine occurs as a neuro-vascular disorder and state of altered brain excitability. Activation and dysfunction of cortical and subcortical networks results in an altered perception of sensory inputs and cause typical neurological symptoms [25].

Recurrent episodic attacks are the hallmark manifestation. Over the course of the attack, various complex and wide-ranging symptoms may occur, owing to the multiple peripheral and central neural networks involved [26]. Activation of the trigeminocervical complex and its projections is thought to contribute to the perception of pain as well as autonomic, cognitive, and affective symptoms [25].

The migraine brain is generally hyperexcitable; neurophysiological studies demonstrate increased excitability to a wide range of nociceptive and non-nociceptive stimuli [27, 28]. This hyperexcitability is thought to contribute to the development of central sensitization which underlies allodynia and peripheral sensitization, and ultimately the throbbing quality and movement sensitivity experienced [29].

Neuroimaging studies reveal that structural and functional changes occur in the migraine brain. Functional magnetic resonance (MR) imaging studies have demonstrated altered connectivity both during attacks and interictal periods. MR spectroscopy and positron emission tomography studies have revealed alterations in energy metabolism and mitochondrial dysfunction in individuals with migraine [30].

#### Functional Neurological Disorders

FND are the manifestation of an incompletely understood pathophysiology and should be conceptualized in a biopsychosocial framework to optimize patient management. Recent work has revealed specific genetic predispositions to the development of FND [31, 32]. Personality and neuropsychological traits, such as neuroticism [33] and alexithymia [34, 35], may play a role. These innate features interact with an individual’s environmental exposures and life experiences, including childhood adversity, as well as medical, neurodevelopmental, neurodegenerative and psychiatric comorbidity [36].

A trigger (identifiable or not) initiates the development of an FND phenotype. Ongoing medical comorbidity and psychosocial stressors can perpetuate symptoms. These predisposing, precipitating, and perpetuating factors disrupt brain network functions involved in sensorimotor processing, impairing the constructs of normal conscious experience. These constructs include attention, salience, emotional processing, interoception, prediction-inference, and importantly, a sense of self agency [37, 38].

As a potential model for the development of FMD, under normal circumstances, the human brain uses a multimodal integration approach, considering internal and external signals, to match a “prediction” of appropriate movement with actual motor cortex output. If the movement does not align with what the feed forward and feedback signals predicted, then the movement will be perceived as involuntary, resulting in the loss of self-agency [38, 39].

Pathophysiology Other FND phenotypes may have overlapping mechanisms. Functional imaging and connectivity studies highlight the involvement of specific brain regions, including the insula, amygdala, and temporoparietal junction, supporting the network-based model of FND [40].

### Possible Associations of FND and Headache

Currently, there is no clearly understood mechanistic intersection of FND and migraine or other headache disorders. There is potentially no true relationship, with comorbid occurrence due to coincidence of two common conditions which could directly or indirectly affect each other.

Yet many authors argue that there may be plausible associations, considering various combinations of neurophysiologic and psychopathologic mechanisms. The concepts and theories to explain a relationship between functional neurologic symptoms and migraine have been discussed per expert opinion (Table 2) [41].

**Table 2:** Proposed possible interactions between functional neurologic symptoms and migraine (*adapted from Stone and Evans 2011 Headache Journal)*

A reversible neurologic dysfunction experienced during a migraine aura could act as a trigger for patients to experience the same type of symptom in a functional manner.
Altered cognition, fatigue, and affective symptoms that may occur in migraine could act as a state in which functional symptoms are more likely to spontaneously occur.
Migraine and FND share multiple comorbidities and may co-occur in the context of a generalized vulnerability to somatic symptoms.
In patients with functional neurologic disorders and migraine, there could be worsening of the functional neurologic symptoms during periods of worsened migraine symptoms.

#### Alterations in Sensory Processing and Brain Network Connectivity

Sensory processing abnormalities may be present in the development of both FND and migraine. Goadsby et al. outline how brainstem and hypothalamic nuclei function as a gate for sensory input. Dysfunction of these nuclei can lead to abnormal reactions to normal sensory stimuli, including the perception of head pain in response to normal blood flow in intracranial vessels or to otherwise “normal” intensities of light, sounds, smell, and touch [25]. There is also evidence of dysfunctional sensory networks during pain-free periods, which is proposed to explain altered sensory processing between migraine attacks [42]. There may be higher sensory processing difficulties in children and adolescents with migraine compared to healthy controls [43, 44]. Findings also support sensory processing difficulties in patients with FND. One study revealed sensory processing tendencies toward low registration, sensory sensitivity, and sensation avoiding [45].

The atypical sensory processing that occurs in both migraine and FND has also been studied within the framework of autism spectrum disorders (ASD). A high proportion of autistic and alexithymic traits has been demonstrated in adults with FND [46]. Only a few studies investigated co-occurrence of migraine and ASD; the two conditions may share common pathophysiological changes [47]. Alexithymia has been identified as more likely to occur in patients with medication overuse headache [48], most of whom have migraine headache disorder. Abnormal sensory processing may trigger paroxysmal functional neurologic symptoms [45]. Similarly, complex sensory input may trigger or aggravate migraine symptoms. Sensory avoidance behavior has been described in both FND and migraine [49]. Interestingly, history of migraine headaches independently predicted individual differences in sensory processing scores in a cohort of FND patients [50].

Imaging studies in FND and migraine provide neuroanatomical considerations for the substrate of overlapping subjective experiences and pathophysiology of these entities. Particularly, the finding of abnormal functional connectivity and variations on volumetric analysis [51–53] of the amygdala [54, 55] and insular cortex [56] in hypersensitivities (to interoceptive [57] and external stimuli), disrupted emotional processing through the limbic system, and salience network dysfunction [58] are seen in both conditions.

#### Shared Comorbid Conditions

Migraine and FND share a range of systemic comorbidities that may occur at higher rates compared to the general population. This includes obesity, sleep disturbances, mood disorders (anxiety, depression), systemic conditions (chronic fatigue, POTS, IBS, hypermobility) and other pain conditions (fibromyalgia) [59–63]. Individually, both migraine and FND are more prevalent in patients who experienced adverse life experiences including childhood abuse or neglect compared with the general population [64, 65].

Possible Associations of FND and Headache Presence of comorbidities may be associated with increased headache frequency, magnitude of disability, and a higher propensity towards central sensitization, leading to a decreased quality of life [66, 67].

###  Functional Neurologic Disorders in Patients with Headache

There are not specific studies looking at rates and characteristics of FND in patients with migraine or other primary headache disorders. There is some literature regarding the occurrence of somatic symptom disorder and new daily persistent headache [68, 69]. Studies regarding the psychiatric comorbidities of migraine do not include mention of functional disorders [66, 70, 71].

Functional Neurologic Disorders in Patients with Headache A 2010 study from India reported a high rate of ‘swooning dissociative’ (non-epileptic) attacks in 23% of 656 unselected female adults with migraine. They report that the attacks typically occurred in the context of migraine. Authors proposed that these findings appeared ‘clearly at odds with published experience’ and that ‘a cultural phenomenon cannot be excluded’ [72].

###  Headache in Patients with Functional Neurologic Disorders 

Current knowledge on the diagnostic rates and occurrence of headache disorder in patients with FND is limited. Within the past five years, further exploration and available data revealed that headache may be a component of the phenotypic complexity for many patients with FND and a prevalent comorbid condition, although rates vary widely.

In an international online survey of FND patients, 70.1% reported headache as a current symptom, 15.4% reported headache in the past, and 14.4% reported never experiencing headache. Headache was the second most reported “associated” symptom, behind fatigue (92.8%). Females constituted 86% of respondents [73].

In a multicenter observational study, migraine was the most frequent coexisting neurological disease in patients with FND (26/410; 6%). FND was more likely to appear after (as opposed to before) the diagnosis of migraine, with a mean latency of over 10 years [74].

Of patients seen at FND clinic, 10% (23/230) had a documented prior or current migraine diagnosis. Migraine was identified as a ‘predisposing,’ ‘precipitating,’ or ‘perpetuating’ factor for many of the participants [75].

In another online survey study, the most reported neurologic comorbidity in patients with FND was migraine (9.6%). Nearly two-thirds of these respondents also reported that migraine diagnosis was retracted after receiving FND diagnosis (decreasing the diagnosis to 3.1%) [59].

#### Psychogenic Non-Epileptic Seizures

A review on migraine in functional (psychogenic non-epileptic) seizures (PNES) was published in this journal in 2017 [76]. We will therefore only highlight below the more recent findings and literature not included.

A recently published meta-analysis investigating potential associations between FND-seizures and migraine and the response of FND-seizures to treatment with migraine prophylactic medications. Overall, the systematic review concluded that all studies found associations between FND-seizures and migraine, which were stronger than those between epileptic seizures and migraine in comparative investigations. There was limited information on the response of FND seizures to treatment with migraine prophylactic medications. In the accompanying case series, investigators reached unanimous consensus that migraine attacks triggered FND-seizures in 28/43 (65.1%); in 73% of patients with adequate follow-up, treatment with migraine prophylactic medications alone (no behavioral interventions) concomitantly reduced FND-seizure and headache frequency by > 50% [77].

Patient- and observer-reported data from 1,372 patients with diagnoses documented by video-electroencephalography compared specific peri-ictal behaviors and seizure triggers among groups with psychogenic and epileptic seizures. Headache aura was more commonly reported in the PNES group [78]. A 2022 study showed the rate of pre-ictal (headache aura) and post-ictal headache was higher in patients with functional seizure events compared to other seizure types [79]. A validated ‘dissociative seizure likelihood score’ which includes history of migraine as associated with PNES can be used as a predictive calculator to distinguish between patients presenting with psychogenic versus epileptic seizures [80, 81].

#### Functional Movement Disorders

In one FMD cohort, 78% (40/51) reported recurrent headache: Of these, 58% met ICHD-3 criteria for migraine or probable migraine, 27% for tension-type headache, 5% for new daily persistent headache, and one for primary exercise headache. The onset of headache onset predated FMD in the majority of patients [82].

Margolesky et al. reported migraine in 62.5% of a cohort of patients with FMD and Ehlers-Danlos syndrome [83]. Tinazzi et al. found migraine to be the most common coexisting neurologic disease in several FMD phenotypes which significantly contributed to disability [74].

In a large questionnaire study, the probability of presence of headache was identified for multiple different FMD symptoms: difficulty walking (0.74), loss of balance (0.76), tremor (0.76), muscle spasms (0.75), muscle jerk (0.74), and trouble swallowing (0.8) or talking (0.75) [73]. Tension headache was reported in 26.4% of patients with facial FMDs, and unilateral facial movements were often accompanied by headache itself [84]. In a multicenter observational study of patients with FMD, headache was reported in 21.2% of those with isolated FMD and 31.9% of those with combined FMD (more than one phenotype) [85].

#### Functional Weakness

A 2007 case control study investigating patients with ‘non-familial migraine with unilateral motor symptoms (MUMS)’ calculated participants’ Conversion V scores (measurement of depression, physical complaints and a combination of traits including denial, the inhibition of emotions, conflict avoidance and possibly conversion disorder). Conversion Vs were more common among patients with MUMS than among controls, but this did not reach statistical significance when corrected for multiple comparisons [86]. Expert opinion highlighted some of the paper’s conclusions, bringing attention to clinical expertise and anecdotal evidence from years of practice, concluding ‘sometimes it is better to err on the generous side when judging the worthiness of symptoms, so‐to‐speak, rather than to jump to the judgment that symptoms not easily explained are just modified madness’ [87].

In one case control study, adults with functional weakness of < 2 years’ duration were more likely to report headache compared to controls (43/107; 40% versus 4/46;9% *p* < 0.0001). Those with functional weakness were also more likely to report neck pain (20/107;19% versus 1/49;2% *p* = 0.005) [88].

In a retrospective interview of this cohort, migraine was found to be an associated symptom in 7% of all cases (8/107). Migraine at onset was more commonly reported in patients who experienced a sudden onset of weakness (< 6 h to maximal onset, as opposed to gradual), migraine was an associated symptom in 10% (5/49). Migraine was less commonly reported at onset compared to panic, dissociative symptoms, pain, and fatigue. Authors concluded that ‘a migrainous motor or sensory symptom, experienced in the context of anxiety, could, through increased attention, persist beyond the immediate migrainous aura’ [89].

In a multicenter observational study, coexistent migraine was reported in 4% (15/410) patients with functional weakness [74]. In a large questionnaire study, patients with limb or facial paralysis had a 0.75 probability of headache [73].

#### Other Functional Symptoms

Functional cognitive disorder refers to complaints of persistent problematic cognitive difficulties, when accompanied by positive features termed ‘internal inconsistency,’ inconsistent with a recognized disease process, and with discrepancies between subjective and observed function [90]. In a large questionnaire study, patients with “memory problems” as FND symptom had a 0.76 probability of headache [73].

Functional sensory symptoms are those in which the patient experiences alteration or absence of normal sensation in the absence of neurologic disease, with the presence of internal inconsistency revealing a pattern of symptoms governed by abnormally focused attention [91]. In a large questionnaire study, patients with numbness or tingling had a 0.75 probability of headache [73].

There is one case report on the occurrence of functional blindness (no organic pathology per examination and work up) in three members of a family, all of whom also experienced severe headache for varying periods of time (days to years); there was resolution of symptoms to varying degrees with suggestion. Authors concluded a functional etiology [92].

Foreign accent syndrome (FAS) is widely understood as an unusual consequence of structural neurological damage but may sometimes represent FND. An observational study of 49 patients with FAS showed migraine as the most common comorbid condition. Migraine or severe headache was the most commonly cited trigger for symptoms (15%), followed by stroke, surgery, and seizure [93].

### Approach to Treatment

 Overall, a clinician’s approach to diagnosis and management may affect outcomes. In one study, participants with NDPH reported value in doctors who displayed interest in each individual case, provided patients with options, allow them to participate, and accompanied them in their plan and process [94]. Meanwhile, in an email survey of headache specialists, 64% marked ‘agree’ or ‘strongly agree’ that patients with functional symptoms often deliberately exaggerate the severity of symptoms [41].

Currently, there is no specific pharmacologic treatment for FND, which is likely a reflection of its elusive and likely heterogeneous pathophysiologic mechanisms [38]. Yet, like migraine, FND is a treatable condition, and patients benefit from specialized and holistic care. Treatment starts with making an accurate diagnosis, conveying this diagnosis to the patient and care team, and ensuring understanding of the diagnosis. Word choice matters; ‘functional,’ as opposed to ‘psychogenic’ or ‘hysterical,’ is preferred terminology [95]. Studies show promise that interventions, including provision of a definitive diagnosis, could reduce this cost (range 9%–90.7%) [2].

A high proportion of patients with FND may receive inadequate migraine prophylacitc or acute treatment [75]. Clinical evaluation of FND patients should include headache in review of symptoms and assessment of the individual’s burden of headache [96]. Appropriate recognition and initial diagnosis of these distinct entities could be critical to outcomes. Managing comorbidities, like migraine, in patients with FND can positively impact the FND symptoms and improve quality of life. In some clinical situations, it may be prudent to distinguish which symptoms are attributed to which disorder.

Recently, non-invasive neurostimulation via transcranial magnetic stimulation (TMS), transcranial direct current stimulation (tDCS) and peripheral nerve stimulation has been studied for FMD treatment [97]. TMS has been shown to be an effective prevention and acute treatment of migraine [98]. Currently, there is only preliminary evidence for application in FMD, and further studies are needed.

#### Multidisciplinary Approaches

It is well-established that both FND and migraine patients may benefit from multidisciplinary and biopsychosocial treatment, and this approach could further favor therapeutic success in patients with both conditions.

For migraine, this should include non-pharmacologic and pharmacologic treatment [99]. ‘Sleep, exercise, eat, diary, and stress has been promoted as the ‘SEEDS’ for success in migraine management [100]. In a recent clinical trial comparing mindfulness-based stress reduction (MBSR) and headache education, both modalities demonstrated improvements in migraine symptoms [101]. Mindfulness-based cognitive therapy for Migraine (MBCT-M) demonstrated efficacy to reduce headache-related disability and attack-level migraine-related disability [102].

For FND, integrated multidisciplinary clinics with neurology, rehabilitation, and psychotherapy may be the optimal approach for substantial symptom improvement [103]. Expert consensus recommendations are available for PT, OT, and ST in the treatment of FND [104–106]. A retrospective cohort study revealed OT improved symptoms in FND patients with sensory processing difficulties [50]. A review on treatment of FND and chronic pain demonstrated that hypnotherapy, cognitive behavioral therapy, and inpatient multidisciplinary treatment with intensive physiotherapy for severe cases have the most evidence to support reduction of symptoms [107]. Other perpetuating factors such as stress, fatigue, anxiety, and sleep disorders should be addressed. Patient management and rehabilitation should be approached from an individualized, tailored basis to optimize QOL and outcomes [108].

## Conclusion

 Migraine frequently coexists with FND, though the reported rates of comorbid occurrence vary widely. Since headache symptoms are not typically part of the core features of FND, headache may not be routinely explored during clinical assessment, which could lead to elevated rates of underdiagnosis and under-reporting in many studies.

There are not specific studies looking at rates and characteristics of FND in patients with migraine or other primary headache disorders. Future research should focus on prospective design studies to elucidate rates of comorbidities. Genetic studies could advance our understanding of the pathophysiological and neurobiological processes underlying FND [31], and identify any shared inheritance patterns and targets for effective treatment.

Migraine and FND share multiple common comorbidities: When present, these co-existing conditions may contribute to greater clinical complexity and suggest potential overlapping mechanisms. These shared comorbidities provide a basis for further research into overlapping pathophysiology between migraine and FND. Shared comorbid condition could help guide the development of optimal treatment approaches grounded in this understanding.

When coexistent, migraine and FND may have significant clinical implications regarding quality of life, disability, and symptom burden. Multidisciplinary, pharmacologic, and non-pharmacologic approaches to treatment should be considered. As headache is identified as a predisposing, precipitating, or perpetuating factor for patients with FND, its early detection and treatment could affect outcomes.

## Key References

- Riva, E., et al., *Beyond movement: Headache in patients with functional movement disorders.* Headache, 2025. 65(1): p. 197–201.
  - ◦ This observational cohort study provides the most recent and thorough investigation of occurrence and phenotype of headache in a cohort of patients with a specific FND phenotype.
- Butler, M., et al., *International online survey of 1048 individuals with functional neurological disorder.* Eur J Neurol, 2021. 28(11): p. 3591-3602.
  - ◦ This international large-scale questionnaire study investigated FND patient characteristics, symptom comorbidities, and illness perceptions.

## Data Availability

No datasets were generated or analysed during the current study.
